# Feature selection and prediction of treatment failure in tuberculosis

**DOI:** 10.1371/journal.pone.0207491

**Published:** 2018-11-20

**Authors:** Christopher Martin Sauer, David Sasson, Kenneth E. Paik, Ned McCague, Leo Anthony Celi, Iván Sánchez Fernández, Ben M. W. Illigens

**Affiliations:** 1 Department of Epidemiology, Harvard T.H. Chan School of Public Health, Boston, MA, United States of America; 2 Institute for Medical Engineering and Science, Massachusetts Institute of Technology, Cambridge, MA, United States of America; 3 Department of Biostatistics, Harvard T.H. Chan School of Public Health, Boston, MA, United States of America; 4 Division of Epilepsy and Clinical Neurophysiology, Department of Neurology, Boston Children’s Hospital, Harvard Medical School, Boston, MA, United States of America; 5 Department of Neurology, Beth Israel Deaconess Medical Center, Harvard Medical School, Boston, MA, United States of America; Zhejiang University, CHINA

## Abstract

**Background:**

Tuberculosis is a major cause of morbidity and mortality in the developing world. Drug resistance, which is predicted to rise in many countries worldwide, threatens tuberculosis treatment and control.

**Objective:**

To identify features associated with treatment failure and to predict which patients are at highest risk of treatment failure.

**Methods:**

On a multi-country dataset managed by the National Institute of Allergy and Infectious Diseases we applied various machine learning techniques to identify factors statistically associated with treatment failure and to predict treatment failure based on baseline demographic and clinical characteristics alone.

**Results:**

The complete-case analysis database consisted of 587 patients (68% males) with a median (p25-p75) age of 40 (30–51) years. Treatment failure occurred in approximately one fourth of the patients. The features most associated with treatment failure were patterns of drug sensitivity, imaging findings, findings in the microscopy Ziehl-Nielsen stain, education status, and employment status. The most predictive model was forward stepwise selection (AUC: 0.74), although most models performed at or above AUC 0.7. A sensitivity analysis using the 643 original patients filling the missing values with multiple imputation showed similar predictive features and generally increased predictive performance.

**Conclusion:**

Machine learning can help to identify patients at higher risk of treatment failure. Closer monitoring of these patients may decrease treatment failure rates and prevent emergence of antibiotic resistance. The use of inexpensive basic demographic and clinical features makes this approach attractive in low and middle-income countries.

## Introduction

Tuberculosis (TB) is a bacterial infectious disease that is endemic in many countries around the world. According to the World Health Organization (WHO), approximately 6.6 million new cases of TB are reported by national and international health organizations each year [1]. The yearly death global toll of TB is estimated to be approximately 1.5 million. Inherently, these figures are underestimations of the true burden of disease since many cases are not diagnosed or reported. Though exact estimates of the true prevalence of TB are variable, most sources recurrently estimate it to exceed 10 million [1, 2]. TB thereby frequently coexists with other infections, such as HIV or hookworms [1]. There are many challenges for effective prevention, diagnosis, and treatment, with some of the most important being lack of funding, limited access to health resources (infrastructure, testing facilities, drug availability), stigmatization, poverty, and lack of compliance. Furthermore, these challenges are highly country dependent, explaining much of the regional variation.

Over the last decade multidrug resistant (MDR) and extensively drug resistant (XDR) TB has become an important public and global health concern. Multidrug resistance is defined as resistance to at least rifampicin and isoniazid, while XDR refers to bacteria that are additionally resistant to any fluoroquinolone and at least one of three injectable second-line drugs (i.e. amikacin, kanamycin, or capreomycin)[2]. While the percentage of MDR/XDR is still relatively small globally, it varies significantly by region and country. In Belarus, the rate of MDR/XDR TB is estimated to be approx. 50/100,000 and has steadily been declining since 2010. Rates exceed 100/100,000 in Thailand and are increasing by approximately 4% annually [1]. Recent data by the WHO estimates the success rate of TB treatment to be approximately 83%, while the success rate for MDR TB is only 54% and even less (30%) for XDR. Promisingly, the introduction of shorter drug regimens has increased treatment success rates to approximately 90% [1].

In a recent publication, Sharma et al. modelled the proportion of TB patients likely to develop MDR/XDR and forecasted a steady increase for most countries over the next 25 years. Particularly alarming rates have been predicted for Russia, where MDR rates might exceed 30% by 2040 [3]. Long established clinical risk factors for treatment failure and subsequent development of MDR/XDR include the number of previous treatments, score on sputum smears, weight and imaging abnormalities (extensive lesions, cavities, mediastinal shift)[4]. More recently, research has focused on the prediction of resistance based on genomic sequencing data. Using whole genome sequencing, Walker et al. identified 120 mutations conferring treatment resistance and were able to predict 89.2% of the validation-set phenotypes with a mean sensitivity of 92.3% and a specificity of 98.4% [5]. Though more accurate than traditional sensitivity essays, it seems unlikely that this technique will be widely applied in low and middle-income countries due to limited resources.

For various conditions, demographics have been established as potent predictors of loss to follow-up [6] and various outcome measures [7]. Here, we aim to explore whether it is possible to predict treatment failure based on basic clinical and demographic information. Various machine learning models were developed to predict treatment failure without making prior assumptions to discover potential novel predictors of treatment failure.

## Methods

### Database

**Fig 1:**
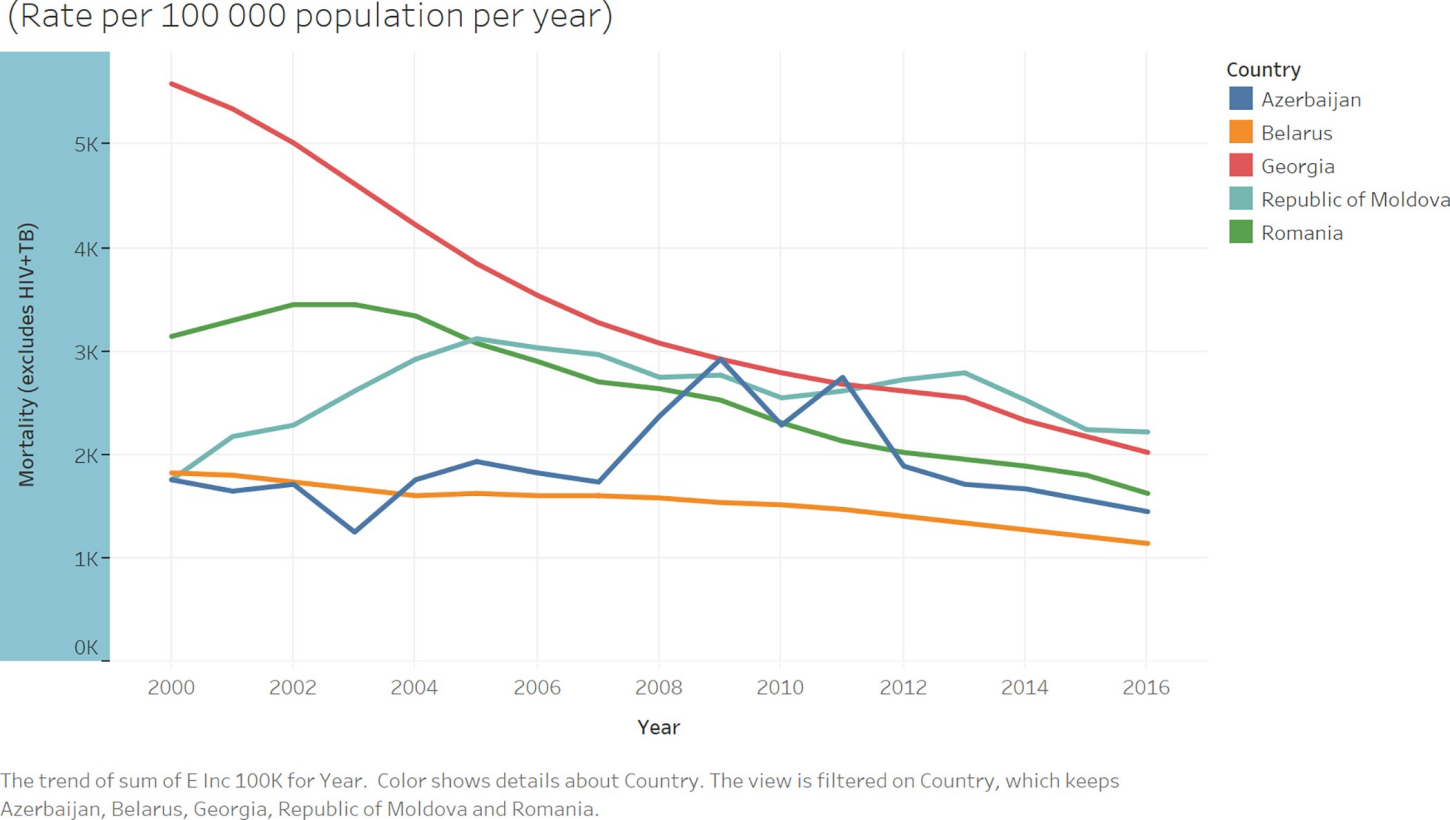
Countries included in the NIAID database and their respective mortality rates. Yearly trends in mortality rates per 100,000 people between 2000 and 2016 are plotted (Data obtained from the WHO Global tuberculosis report 2017 [1]).

The National Institute of Allergy and Infectious Diseases (NIAID) Office of Cyber Infrastructure and Computational Biology (OCICB) established the TB Portals Program as a multi-national collaboration for TB data sharing and analysis to advance TB research [8]. A consortium of clinicians and scientists from countries with a heavy burden of TB, especially drug-resistant TB, work together with data scientists and IT professionals to collect multidomain TB data and make it available to the clinical and research communities. The majority of cases in the TB Portals is from drug-resistant TB. The TB Portals dataset includes clinical, imaging, genomic, and other metadata integrated in patient case records, which originate from countries around the world. This study analyzed data from Azerbaijan, Belarus, Moldova, Georgia, and Romania (Fig 1). The main descriptive features of the database are presented in Table 1.

**Table 1:** Basic demographic and clinical features of the study population stratified by treatment outcome. BMI: Body mass index, MDR: multi-drug resistant, XDR: extensively drug resistant.

Variables	Level	Treatment success	Treatment failure
Number of participants (n (%))		491 (76.36%)	152 (23.64%)
Female (n (%))		168 (34.2%)	35 (23.0%)
BMI (mean (±sd))		21.31 (±3.52)	21.04 (±3.01)
Age of onset (mean (±sd))		41.11 (±14.78)	42.56 (±13.57)
Country (n (%))	Azerbaijan	51 (10.4%)	9 (5.9%)
	Belarus	307 (62.5%)	106 (69.7%)
	Georgia	29 (5.9%)	31 (20.4%)
	Moldova	15 (3.1%)	3 (2.0%)
	Romania	89 (18.1%)	3 (2.0%)
Type of resistance (n (%))	Sensitive	95 (19.3%)	11 (7.2%)
	Mono drug resistant	26 (5.3%)	1 (0.7%)
	Poly drug resistant	8 (1.6%)	6 (3.9%)
	MDR	269 (54.8%)	89 (58.6%)
	XDR	89 (18.1%)	44 (28.9%)
	Not reported	4 (0.8%)	1 (0.7%)
Employment (n (%))	Disabled	29 (5.9%)	18 (11.8%)
	Employed	187 (38.1%)	36 (23.7%)
	Not reported	21 (4.3%)	2 (1.3%)
	Retired	47 (9.6%)	10 (6.6%)
	Student	23 (4.7%)	1 (0.7%)
	Unemployed	184 (37.5%)	85 (55.9%)
Social risk factors (n(%))	Alcoholism	89 (18.1%)	36 (23.7%)
	Current smoker	181 (36.9%)	69 (45.4%)
	Documented MDR contact	10 (2.0%)	1 (0.7%)
	Ex-prisoner	3 (0.6%)	2 (1.3%)
	Homeless	1 (0.2%)	0 (0.0%)
	Not reported	204 (41.5%)	43 (28.3%)
	Registered drug abuse	1 (0.2%)	0 (0.0%)
	Worked abroad	2 (0.4%)	1 (0.7%)
Education (n (%))	Primary schooling	38 (7.7%)	6 (3.9%)
	College bachelor	170 (34.6%)	56 (36.8%)
	High School	165 (33.6%)	47 (30.9%)
	Higher University	66 (13.4%)	10 (6.6%)
	Not reported	52 (10.6%)	33 (21.7%)
Regimen drug (n (%))	Amikacin	51 (10.4%)	20 (13.2%)
	Amoxicillin-clavulanate	62 (12.6%)	35 (23.0%)
	Bedaquiline	29 (5.9%)	2 (1.3%)
	Capreomycin	146 (29.7%)	56 (36.8%)
	Clofazimine	3 (0.6%)	0 (0.0%)
	Cyclosporine	71 (14.5%)	12 (7.9%)
	Ethambutol	113 (23.0%)	17 (11.2%)
	Not reported	6 (1.2%)	2 (1.3%)
Total cavity formation (n (%))	No cavities	173 (35.2%)	52 (34.2%)
	1 cavity	76 (15.5%)	34 (22.4%)
	2 cavities	31 (6.3%)	13 (8.6%)
	>2 cavities	46 (9.4%)	36 (23.7%)
	Not reported	165 (33.6%)	17 (11.2%)
Size of cavities (n (%))	No cavities	173 (35.2%)	52 (34.2%)
	>2 5mm	29 (5.9%)	35 (23.0%)
	<10 mm	87 (17.7%)	26 (17.1%)
	10–25 mm	37 (7.5%)	22 (14.5%)
	Not reported	165 (33.6%)	17 (11.2%)
Calcified nodes (n (%))	No	280 (57.0%)	109 (71.7%)
	Yes	46 (9.4%)	26 (17.1%)
	Not reported	165 (33.6%)	17 (11.2%)
Decreased lung capacity (n (%))	No	239 (48.7%)	77 (50.7%)
	Not reported	163 (33.2%)	17 (11.2%)
	Yes	89 (18.1%)	58 (38.2%)
Pneumothorax (n (%))	No	318 (64.8%)	131 (86.2%)
	Yes	9 (1.8%)	4 (2.6%)
	Not reported	164 (33.4%)	17 (11.2%)
Dissemination (n (%))	No	242 (49.3%)	94 (61.8%)
	Not reported	160 (32.6%)	17 (11.2%)
	Yes	89 (18.1%)	41 (27.0%)
Shadow pattern (n (%))	Infiltrates	11 (2.2%)	8 (5.3%)
	Node >10 mm	4 (0.8%)	2 (0.3%)
	Nodule and node	147 (29.9%)	49 (32.2%)
	Nodule <10 mm	77 (15.7%)	33 (21.7%)
	Nodule, node and infiltrate	85 (17.3%)	43 (28.3%)
	Not reported	167 (34.0%)	17 (11.2%)
Number of CTs (mean (sd))		1.64 (1.58)	1.09 (1.09)
Number of daily contacts (mean (sd))		2.21 (1.62)	2.13 (1.72)
Lung localization (n (%))	Not reported	3 (0.6%)	0 (0.0%)
	Pulmonary	345 (70.3%)	78 (51.3%)
	Pulmonary and extrapulmonary	143 (29.1%)	74 (48.7%)
Number of X-rays (mean (sd))		1.98 (2.13)	1.93 (2.50)
Process prevalence (n (%))	≥2 segments	208 (42.4%)	108 (71.1%)
	<2 segments	118 (24.0%)	27 (17.8%)
	Not reported	165 (33.6%)	17 (11.2%)
Pleuritis (n (%))	No	306 (62.3%)	121 (79.6%)
	Not reported	164 (33.4%)	17 (11.2%)
	Yes	21 (4.3%)	14 (9.2%)

In this study we used data from the TB portals (https://depot.tbportals.niaid.nih.gov), which has been stripped of all individual identifying information and has been assigned random, unique codes. While the database continues to be actively populated with participants, we used all available data as per March 2018. NIH safeguards the confidentiality and privacy of the individuals whose data have been deposited into the TB Portals. The primary outcome was treatment failure, which we defined as failure of therapy or death. The variables used for prediction were: country, age of onset, sex, education level, employment status, number of daily contacts, type of resistance, body mass index (BMI), localization in the lung, number of X-rays, number of CT scans, dissemination (diffuse pulmonary nodules detected), size of the lung cavity, pleural involvement, imaging pattern, pneumothorax, pleuritis, nodal calcinosis, process extension, decrease in lung capacity, lung caverna, culture results, microscopy results, social risk factors (including smoking, alcoholism, ex-prisoner, etc.), and drug regimen. Imaging features were based mostly on chest CT scans, except for Georgia, where features were based mostly on chest X-rays.

### Dataset division

We randomly split the original dataset into 70% of patients for a training subset and 30% for a testing or validation subset. The clinical characteristics of the training and testing subsets were similar (S1 Table). Predictive models were developed using only the training subset data and, only after all predictive models were developed, were they tested on the testing data subset. The performance of the models was optimized with cross-validation in the training subset before implementing them in the testing subset. This approach ensures that the performance of the algorithms in the testing subset is similar to their performance in other data which the models have never been exposed to.

We performed a complete case analysis with only the cases that had all variables available. As a sensitivity analysis, we performed the same analysis with all cases after imputing missing values with multiple imputation using predictive mean matching. The limits between statistics and machine learning are difficult to delineate [9]. For the purpose of this study we considered machine learning techniques as those that developed models based only on the data available without any human intervention. We used several of the most commonly used techniques for machine learning: stepwise forward selection, stepwise backward elimination, backward elimination and forward selection, Least Absolute Shrinkage and Selection Operator (LASSO) regression, random forests, support vector machine (SVM) with linear kernel and polynomial kernel [10, 11]. The stepwise selection or elimination models select variables based on a greedy algorithm that tries to minimize the Akaike Information Criterion (AIC) and, therefore, balances predictive power with model complexity [10, 11]. LASSO regression also penalizes model complexity but tends to shrink less important variables towards zero, so that the resulting model is sparse and more interpretable. Random forests fits several de-correlated decision trees to the data and “averages” them so that the resulting model has as little bias and as little variance as possible [10, 11]. With de-correlation only a random subset of all available variables is used for each given branch, ensuring that the individual trees are heterogeneous and capture as much variability in the data as possible. As observations might not be separable in their original dimensional space, support vector machines separate the observations in a higher dimensional space that they visit through a kernel [10, 11]. Regarding parameter tuning, forward stepwise selection, backwards stepwise elimination, and backwards elimination with forward selection did not require parameter tuning and just minimized the AIC. In LASSO regression, the lambda value that shrinks small coefficients to zero was chosen by cross-validation in the training set [10, 11]. For random forests, we selected 1000 trees, a commonly used default, and the default leaf size of 5 in the randomForest function in R. We selected the number of variables randomly sampled as candidates at each split (mtry parameter in the randomForest function in R) by cross-validation in the training set. In SVM, the cost parameter quantifies the cost of a violation of the margin: when the cost is small the margins will be wide and many support vectors will be on the margin or will violate the margin, in contrast, when the cost is large the margins will be narrow and there will be few support vectors on the margin or violating the margin [11]. The cost was chosen by cross-validation in the training set. The gamma parameter for non-linear kernels in SVM is the free parameter of the Gaussian radial basis function. Large gamma leads to high bias and low variance models and vice versa. We used the default value of 1/data dimension in the SVM function in R for the gamma parameter. In summary, we used defaults for the most established parameters and tuned the rest of the parameters with cross-validation in the training set. Predictive performance was measured with the area under the receiving operating characteristic curve (AUC) in the testing model. The thresholds for prediction in an AUC are domain-specific and depend on the trade-off between the consequences of false positives and false negatives. We calculated the statistically “optimal” cut point in the probability of treatment failure: the Youden index, which maximizes sensitivity–(1 –specificity) [12]. Based on these thresholds, in order to give an approximate idea of clinical applicability, we provide sensitivities, specificities, positive predictive values (PPV), and negative predictive values (NPV) in our cohort. The full code for performing this study with full results is available on github (www.github.com/dsasson48/niaid_TB).

### Statistical software

All statistical analyses were performed with R (version 3.2.2) and the packages gmodels, caTools, MASS, glmnet, randomFores, e1071, mice, gridExtra, ggplot2, and pROC. WHO data were visualized with Tableau Desktop (version 10.3, Tableau, Seattle, WA, USA).

## Results

### Demographic and clinical features

The complete-case analysis database consisted of 587 patients (68% males) (411 for training and 176 for testing) with a median (p25-p75) age of 40 (30–51) years. Belarus contributed a major proportion of cases. Treatment failure occurred in approximately one fourth of the cases. The demographic and clinical features are summarized in Table 1.

### Feature selection

**Fig 2:**
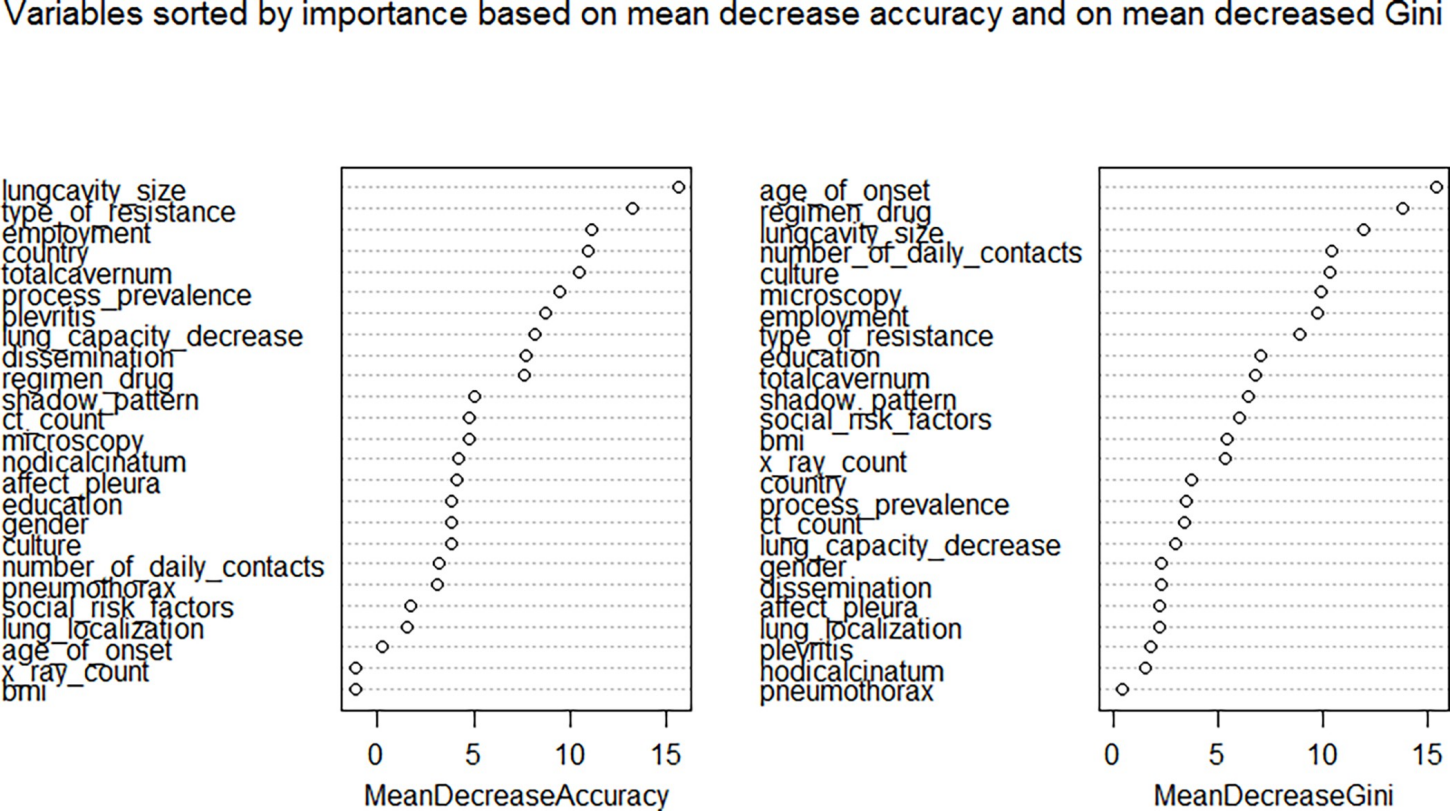
**Variable importance in random forests considering mean decrease in accuracy (left) or mean decrease in Gini index (right).** In random forest models, node heterogeneity can be measured as a decrease in classification accuracy over all out-of-bag validated predictions when a variable is permuted after training and before prediction (mean decrease accuracy) or it can be measured as a decreased in node impurity (mean decreased Gini). The term “decrease” in these metrics does not imply any direction of the correlation.

Features that were statistically significantly most associated with the outcome of treatment failure are summarized in Table 2. The patterns of drug sensitivity, imaging findings, drug regimen, education and employment were associated with the outcome of treatment failure. As an illustrative example, the importance of variables for the random forest model is shown in Fig 2.

**Table 2:** Main factors correlated with treatment failure. LASSO: Least absolute shrinkage and selection operator.

Model	Top factors correlated with treatment failure
**Forward stepwise selection**	Negatively correlated: drug sensitivity (sensitive), employment status (employed), microscopy: 1 to 99 acid-resistant bacteria in 100 fields of view when stained by Ziehl-Nielsen, dissemination (diffuse pulmonary nodules detected)
**Backward stepwise elimination**	Negatively correlated: employment status (employed), drug sensitivity (sensitive), dissemination (diffuse pulmonary nodules detected), microscopy: 0 acid-resistant bacteria in 100 fields of view when stained by Ziehl-Nielsen
**Backwards elimination and forward stepwise selection**	Negatively correlated: employment status (employed), drug sensitivity (sensitive), dissemination (diffuse pulmonary nodules detected), microscopy: 0 acid-resistant bacteria in 100 fields of view when stained by Ziehl-Nielsen
**LASSO**	Positively correlated: country (Georgia), employment status (unemployed), extrapulmonary localization, lung cavity size (more than 25mm), decrease in lung capacity, microscopy: more than 99 acid-resistant bacteria in 100 fields of view when stained by Ziehl-Nielsen Negatively correlated: country (Romania), employment status (employed), employment status (student), drug sensitivity (sensitive), pattern of chest imaging (not reported)
**Random forests** **(see Fig 2)**	Unknown whether positively or negatively correlated: By mean decrease in accuracy: lung cavity size, type of resistance, employment status, country, total caverna By mean decrease in Gini index: age of onset, drug regimen, lung cavity size, number of daily contacts

### Prediction performance

**Fig 3:**
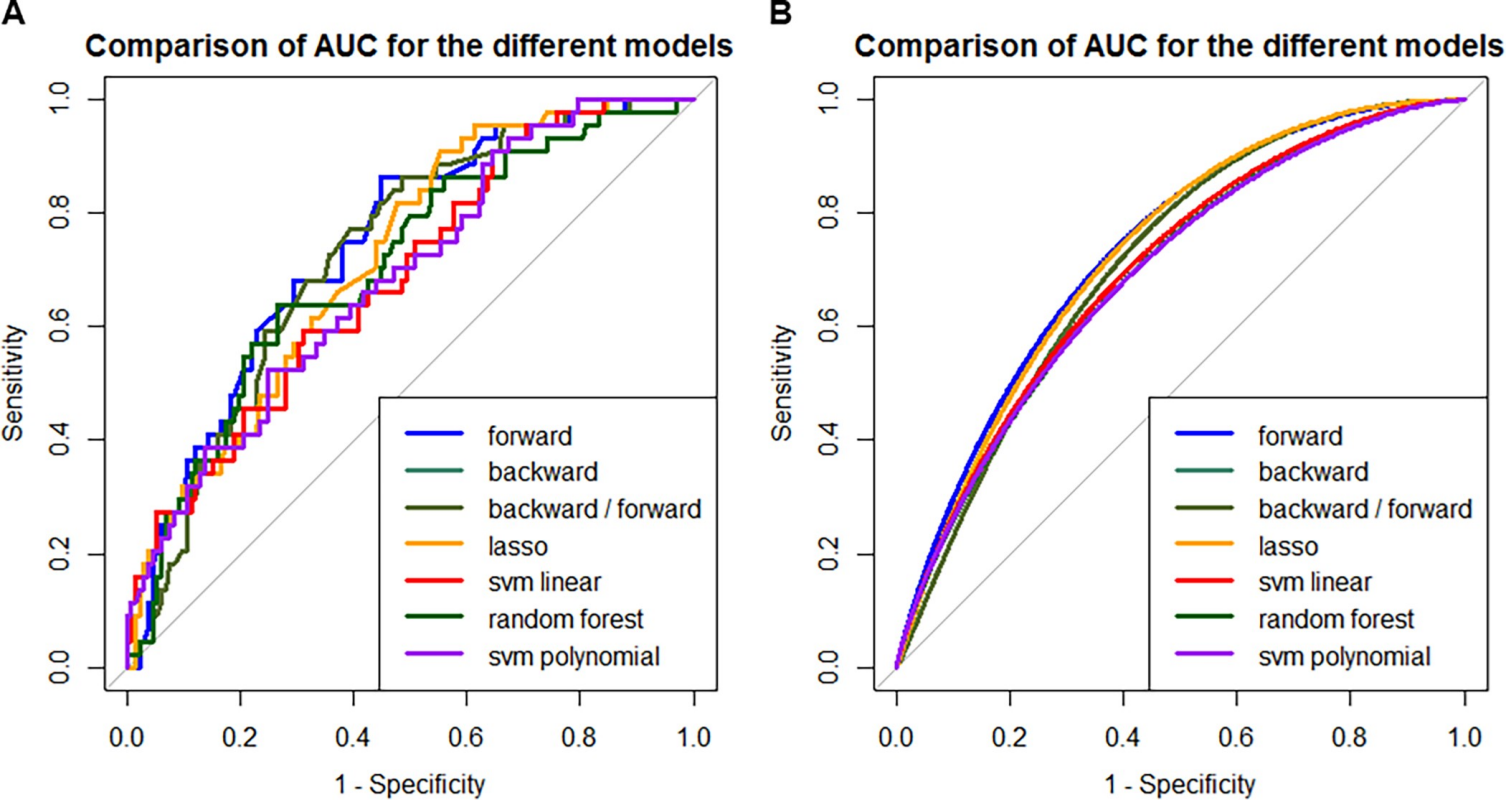
**Comparison of (A) non-smooth and (B) smooth AUC for the different models.** Linear models outperform the other machine learning models.

We compared the prediction performance of the different machine learning models in their ability to predict treatment failure. The most predictive model was forward stepwise selection, although most models performed at or above AUC 0.7 (Table 3, Fig 3). If predictive models are used as a screening tool for identifying subjects that may benefit from more detailed evaluation of resistant TB, forward stepwise selection was the best model with a sensitivity of 0.36 and a NPV of 0.81. If predictive models are used to start treatment in resistant TB, the best model was LASSO with a specificity of 0.96 and a PPV of 0.64. Depending on the intended use of predictive models, modifications of the thresholds may provide higher sensitivities and specificities that meet the intended use of the model.

**Table 3:** Comparison of the prediction performance of the different statistical models. Classic regression approaches with forward and/or backward stepwise selection yield the highest AUC. Exemplary values for misclassification, sensitivity, specificity, PPV and NPV are provided for reference. AUC: Area under the receiver-operator curve, 95% CI: 95% Confidence interval, PPV: Positive predictive value, NPV: Negative predictive value, LASSO: Least absolute shrinkage and selection operator, SVM: Support vector machine.

Method	AUC (95% CI)	Misclassi-fication	Sensitivity	Specificity	PPV	NPV
**Forward stepwise selection**	0.74 (0.66–0.82)	0.24	0.36	0.89	0.53	0.81
**Backward stepwise elimination**	0.73 (0.65–0.81)	0.27	0.3	0.88	0.45	0.79
**Backward stepwise elimination & forward stepwise selection**	0.73 (0.65–0.81)	0.27	0.30	0.88	0.45	0.79
**LASSO**	0.72 (0.64–0.80)	0.23	0.21	0.96	0.64	0.78
**Random forest**	0.70 (0.62–0.79)	0.24	0.30	0.91	0.52	0.80
**SVM linear kernel**	0.69 (0.60–0.77)	0.24	0.21	0.94	0.56	0.78
**SVM polynomial kernel**	0.69 (0.60–0.77)	0.25	0	1	NA	0.75

### Sensitivity analysis

To evaluate the robustness of our findings, we performed the same analyses in the dataset considering all original patients and imputing missing values with multiple imputation. This database of 643 patients (450 patients in the training set and 193 patients in the testing set) showed similar demographic and clinical features than the complete case analysis dataset. The factors consistently associated with treatment failure persisted to be the patterns of drug sensitivity, imaging findings, drug regimen, education and employment and some other factors like culture findings, or number of daily contacts. The predictive performance in the imputed dataset was generally superior than in the complete case analysis dataset, suggesting that larger datasets may improve prediction power (S2 Table and S1 Fig).

## Discussion

This study identified patterns of drug sensitivity, imaging findings, findings in the microscopy Ziehl-Nielsen stain, education status, and employment status as the top factors correlated with treatment failure. In addition, our models based on basic demographic and clinical features predicted treatment failure with an AUC at or above 0.7, with the best-performing model having an AUC of 0.74.

The first to use clinical and demographic data for the prediction of tuberculosis treatment were Keane et al. in 1997, who compared 130 Vietnamese subjects failing to respond to 673 responders. Hereby, they identified X-ray signs and degree of sputum smear positivity as the strongest predictors of treatment failure (70% sensitivity, 80% specificity) [4]. In 2002, studying 676 patients from Southern India, Santha et al. identified irregular treatment, being male, alcoholism and history of previous therapy to be associated with treatment failure [13]. More recently, Huang and colleagues reported extensive lesions, cavities and mediastinal shift to have the strongest association with treatment failure in their cohort [14]. Furthermore, they established age above 60 years and a history of smoking to be predictive. Concurrently, a case series of 167 TB patients in Iran identified coinfection with other pathogens, male sex, family history of TB, Turkoman ethnicity, and household size to be associated with treatment failure [15]. Of note, most studies reported different factors to have the strongest predictive performance. These differences are likely due to considerable heterogeneity in the underlying databases, regional population demographics and divergent analysis approaches. Furthermore, some studies were relatively small, thus allowing factors to become strong predictors by chance.

The first to employ a logistic regression model for prediction were Kalhori et al. in 2008, which, using clinical data, achieved an AUC of 0.70 [16]. Similarly, Rodrigo et al. report a predictive scoring instrument for tuberculosis lost to follow-up with an AUC of 0.67 (95%CI 0.65–0.70) [17]. Recently, Cherkaoui et al. conducted a prospective study employing questionnaires in rural Morocco. In the post-hoc analysis, the authors developed a scoring system with a high AUC (0.93, 95%CI 0.90–0.96), which was however not been validated in either a validation arm or external dataset and is therefore likely to be an overestimate of the true predictive power [18]. Though our dataset is relatively small, our unsupervised machine-learning approach still yielded a predictive model that outperformed previous ones.

Apart from emphasizing the importance of several well-established microbiological and imaging factors, our results add to the existing literature by identifying demographic and clinical factors with a strong and consistent statistical association with treatment failure or death, such as level of education and employment status. These results come from the application of machine learning techniques on data to discover associations that might have been overlooked by hypothesis-driven approaches. At the same time, both level of education and employment status are appealing predictors to treatment response, since they have been shown to affect health in various ways, including but not limited to health care seeking, treatment initiation, coherence and response in various other diseases [19–22]. Furthermore, the efficiency of TB treatment provision depends on national policies and health care systems, thus explaining why country is a strong predictor in our model. Number of daily contacts is strongly correlated with health care seeking behavior and treatment complications, all of which have been shown to predict outcome [23].

The identification and validation within a predictive model allows for easy and cheap identification of patients at high risk for treatment failure and can be used as a starting point for a patient-centered approach to resistance prevention.

### Machine learning

Learning from data is typically used in situations with no theoretical or prior knowledge solution, but where data can be used to construct an empirical solution [10]. Currently most regression models in medicine consist of multivariable linear or logistic regression models constructed based on biological plausibility and prior medical knowledge. These models emphasize causality and have the major advantage of simplicity and interpretability: it is clear from the model which variables influence the outcome and their relative weights. In contrast, the more complex the machine learning models, the less intuitive interpretation of results [9]. In this study, we show that machine learning algorithms provided additional information as compared to explanatory models in prior literature based on prior medical knowledge. Furthermore, it had a higher AUC than any previous studies relying on clinical or demographic data.

Recently, many specialties including public health, are experiencing an increase in multicenter collaborations that yield datasets with large numbers of variables collected on numerous patients. The rapid increase in the number and quality of large datasets makes it possible to implement big data approaches to data analysis in the field of public health and TB.

Recently, a wide and deep neural network used targeted or whole genome sequencing and conventional drug resistance phenotyping data from 3,601 *M*. *tuberculosis* strains, 1,228 of which were multidrug resistant, to predict phenotypic drug resistance to 10 anti-tubercular drugs [24]. On an independent validation set, this wide and deep neural network showed significant performance gains over baseline models, with average sensitivities and specificities, respectively, of 84.5% and 93.6% for first-line drugs and 64.0% and 95.7% for second-line drugs [24].

In this publication, we relied on clinical data rather than genomic data, since gene sequencing is expensive and not widely available in low and middle-income countries. Contrarily, clinical data is easily accessible and can be used without incurring additional costs.

### Strengths and weaknesses

The present study demonstrates that machine learning techniques add to explanatory models by identifying variables that may be correlated with TB treatment failure, thus informing better predictive models. All machine learning techniques, which have no subject-matter knowledge, identified relevant variables and showed a consistently high predictive performance.

The advantage of our model compared to pharmacological [25], microbiological [26] or genetic approaches [5, 27] is that the required data is already been collected routinely, thus being widely available and cheap, which is a major advantage in resource- and budget-strained environments.

The clinical and public health contribution of this study are to provide low- and middle-income countries with inexpensive and easy to apply tools to identify populations where the yield of identification and treatment of resistant TB may be highest. Different stakeholders may build on our analyses, for which we provide the full code online.

Importantly, we do not intend to imply that factors correlated with resistant TB such as level of education, or level of employment are pathophysiologically causal in resistance to antituberculotic medications. Drug resistance is ultimately dependent on factors of the causal germ: Mycobacterium tuberculosis. A detailed microbiological and genomic characterization of the strain of Mycobacterium tuberculosis from each patient may predict resistant TB better [24], but is frequently not be feasible in low-resource setting. The orientation of this manuscript therefore was to find demographic and clinical features that are inexpensive to collect and can predict TB treatment failure. These factors may help identify inexpensively populations where the yield of detecting and treating resistant TB may be higher. The purpose of this manuscript was not to improve the performance of well-established algorithms in machine learning, but to show that different machine learning algorithms may be used to predict resistant TB using widely available data and off-the-shelf machine learning algorithms.

Missing data may have influenced the predictive capacity of our models. The sensitivity analysis imputing missing data showed an increased performance compared to the complete case analysis, although these results may also be interpreted as the higher predictive performance of a larger dataset. Furthermore, combining treatment failure and death might have biased the results towards the null since some cases might have died independent of tuberculosis (e.g. from traumatic causes). Another limitation is that the underlying data is derived from only a few countries in Eastern Europe.

Future studies in larger datasets should therefore validate our findings and prediction models for other countries. The current dataset was collected with a focus on resistant TB. Therefore, patients with resistant TB cases are oversampled in this dataset and generalizability might therefore be limited to countries with high resistance rates. In the future, addition of more variables and cases to the NIAID tuberculosis dataset might further improve predictive performance. Additional studies could focus on translation of these findings into a validated (online) prediction model that can be used by clinicians to identify patients at highest risk of treatment failure in routine practice.

## Conclusions

In a mid-size dataset we found that machine learning techniques added information to explanatory models for prediction of resistance to TB treatment. The novel identification of demographic factors and validation of a prediction tool for TB treatment failure provides a proof-of-concept that use of routinely collected data can help to improve routine care. This approach can potentially be more cost-effective in resource-constrained environments.

## Supporting information

S1 Table Comparison of patient characteristics in the training and testing subset. IQR: interquartile range.
(DOCX)

S2 Table Comparison of prediction performance of the different models in the imputed dataset. Predictive performance is higher than in the complete cases analysis (Table 3). AUC: Area under the receiver-operator curve, PPV: Positive predictive value, NPV: Negative predictive value, LASSO: Least absolute shrinkage and selection operator, SVM: Support vector machine.
(DOCX)

S1 Fig Predicted (A) and smoothed (B) AUC for the different models in the imputed dataset.
(TIF)

## References

[1] WHO. Global tuberculosis report 2017 2017. Available from: http://apps.who.int/iris/bitstream/10665/259366/1/9789241565516-eng.pdf.

[2] CDC. Fact Sheets | Drug-Resistant TB | Extensively Drug-Resistant Tuberculosis (XDR TB) | TB | CDC 2018 [updated 20 Feb 2018]. Available from: https://www.cdc.gov/tb/publications/factsheets/drtb/xdrtb.htm.

[3] Sharma A, Hill A, Kurbatova E, van der Walt M, Kvasnovsky C, Tupasi TE, et al Estimating the future burden of multidrug-resistant and extensively drug-resistant tuberculosis in India, the Philippines, Russia, and South Africa: a mathematical modelling study. Lancet Infect Dis. 2017;17(7):707–15. Epub 2017/05/14. 10.1016/S1473-3099(17)30247-5 ; PubMed Central PMCID: PMCPMC5599934. 28499828 10.1016/S1473-3099(17)30247-5 PMC5599934

[4] Keane VP, de Klerk N, Krieng T, Hammond G, Musk AW. Risk factors for the development of non-response to first-line treatment for tuberculosis in southern Vietnam. Int J Epidemiol. 1997;26(5):1115–20. Epub 1997/11/18. . 9363535 10.1093/ije/26.5.1115

[5] Walker TM, Kohl TA, Omar SV, Hedge J, Del Ojo Elias C, Bradley P, et al Whole-genome sequencing for prediction of Mycobacterium tuberculosis drug susceptibility and resistance: a retrospective cohort study. Lancet Infect Dis. 2015;15(10):1193–202. Epub 2015/06/28. 10.1016/S1473-3099(15)00062-6 ; PubMed Central PMCID: PMCPMC4579482. 26116186 10.1016/S1473-3099(15)00062-6 PMC4579482

[6] Sielatycki JA, Parker SL, Godil SS, McGirt MJ, Devin CJ. Do Patient Demographics and Patient-Reported Outcomes Predict 12-Month Loss to Follow-Up After Spine Surgery? Spine (Phila Pa 1976). 2015;40(24):1934–40. Epub 2015/11/26. 10.1097/brs.0000000000001101 . 26595443 10.1097/BRS.0000000000001101

[7] Wilson D, Jin DL, Wen T, Carmichael JD, Cen S, Mack WJ, et al Demographic factors, outcomes, and patient access to transsphenoidal surgery for Cushing's disease: analysis of the Nationwide Inpatient Sample from 2002 to 2010. Neurosurg Focus. 2015;38(2):E2 Epub 2015/02/03. 10.3171/2014.11.FOCUS14694 . 25639320 10.3171/2014.11.FOCUS14694

[8] Rosenthal A, Gabrielian A, Engle E, Hurt DE, Alexandru S, Crudu V, et al The TB Portals: an Open-Access, Web-Based Platform for Global Drug-Resistant-Tuberculosis Data Sharing and Analysis. J Clin Microbiol. 2017;55(11):3267–82. Epub 2017/09/15. 10.1128/JCM.01013-17 ; PubMed Central PMCID: PMCPMC5654911. 28904183 10.1128/JCM.01013-17 PMC5654911

[9] Beam AL, Kohane IS. Big Data and Machine Learning in Health Care. Jama. 2018;319(13):1317–8. Epub 2018/03/14. 10.1001/jama.2017.18391 . 29532063 10.1001/jama.2017.18391

[10] Abu-Mostafa YS M-IM, Lin H-T. Learning from Data: A Short Course 2012.

[11] James G WD, Hastie T, Tibshirani R. An Introduction to Statistical Learning: with Applications in R: Springer Science & Business Media; 2013.

[12] Perkins NJ, Schisterman EF. The inconsistency of "optimal" cutpoints obtained using two criteria based on the receiver operating characteristic curve. Am J Epidemiol. 2006;163(7):670–5. Epub 2006/01/18. 10.1093/aje/kwj063 ; PubMed Central PMCID: PMCPMC1444894. 16410346 10.1093/aje/kwj063 PMC1444894

[13] Santha T, Garg R, Frieden TR, Chandrasekaran V, Subramani R, Gopi PG, et al Risk factors associated with default, failure and death among tuberculosis patients treated in a DOTS programme in Tiruvallur District, South India, 2000. Int J Tuberc Lung Dis. 2002;6(9):780–8. Epub 2002/09/18. . 12234133

[14] Huang Q, Yin Y, Kuai S, Yan Y, Liu J, Zhang Y, et al The value of initial cavitation to predict re-treatment with pulmonary tuberculosis. Eur J Med Res. 2016;21(1):20 Epub 2016/05/08. 10.1186/s40001-016-0214-0 ; PubMed Central PMCID: PMCPMC4858857. 27154410 10.1186/s40001-016-0214-0 PMC4858857

[15] Mohammadzadeh Kh A, Ghayoomi A, Maghsoudloo D. Evaluation of factors associated with failure of tuberculosis treatment under DOTS in northern Islamic Republic of Iran. East Mediterr Health J. 2016;22(2):87–94. Epub 2016/05/18. . 27180736 10.26719/2016.22.2.87

[16] Sharareh R. Niakan Kalhori MN, Zeng Xiao-Jun. A Logistic Regression Model to Predict High Risk Patients to Fail in Tuberculosis Treatment Course Completion. International Journal of Applied Mathematics. 2008.

[17] Rodrigo T, Caylà JA, Casals M, García-García JM, Caminero JA, Ruiz-Manzano J, et al A predictive scoring instrument for tuberculosis lost to follow-up outcome. Respiratory Research. 2012;13(1):75 10.1186/1465-9921-13-75 22938040 10.1186/1465-9921-13-75 PMC3490987

[18] Cherkaoui I, Sabouni R, Ghali I, Kizub D, Billioux AC, Bennani K, et al Treatment Default amongst Patients with Tuberculosis in Urban Morocco: Predicting and Explaining Default and Post-Default Sputum Smear and Drug Susceptibility Results. PLOS ONE. 2014;9(4):e93574 10.1371/journal.pone.0093574 24699682 10.1371/journal.pone.0093574 PMC3974736

[19] Kondo N, Sembajwe G, Kawachi I, van Dam RM, Subramanian SV, Yamagata Z. Income inequality, mortality, and self rated health: meta-analysis of multilevel studies. Bmj. 2009;339:b4471 Epub 2009/11/12. 10.1136/bmj.b4471 ; PubMed Central PMCID: PMCPMC2776131. 19903981 10.1136/bmj.b4471 PMC2776131

[20] Subramanian SV, Kawachi I. Income inequality and health: what have we learned so far? Epidemiol Rev. 2004;26:78–91. Epub 2004/07/06. 10.1093/epirev/mxh003 . 15234949 10.1093/epirev/mxh003

[21] Lynch J, Smith GD, Harper S, Hillemeier M, Ross N, Kaplan GA, et al Is income inequality a determinant of population health? Part 1. A systematic review. Milbank Q. 2004;82(1):5–99. Epub 2004/03/16. 10.1111/j.0887-378X.2004.00302.x ; PubMed Central PMCID: PMCPMC2690209. 15016244 10.1111/j.0887-378X.2004.00302.x PMC2690209

[22] Marmot MG, Smith GD, Stansfeld S, Patel C, North F, Head J, et al Health inequalities among British civil servants: the Whitehall II study. Lancet. 1991;337(8754):1387–93. Epub 1991/06/08. . 1674771 10.1016/0140-6736(91)93068-k

[23] Khan MS, Hutchison C, Coker RJ. Risk factors that may be driving the emergence of drug resistance in tuberculosis patients treated in Yangon, Myanmar. PLoS ONE. 2017;12(6):e0177999 10.1371/journal.pone.0177999 PMC5470668. 28614357 10.1371/journal.pone.0177999 PMC5470668

[24] Chen ML, Doddi A, Royer J, Freschi L, Schito M, Ezewudo M, et al Deep Learning Predicts Tuberculosis Drug Resistance Status from Whole-Genome Sequencing Data. bioRxiv. 2018.

[25] Sloan DJ, Mwandumba HC, Garton NJ, Khoo SH, Butterworth AE, Allain TJ, et al Pharmacodynamic Modeling of Bacillary Elimination Rates and Detection of Bacterial Lipid Bodies in Sputum to Predict and Understand Outcomes in Treatment of Pulmonary Tuberculosis. Clin Infect Dis. 2015;61(1):1–8. Epub 2015/03/18. 10.1093/cid/civ195 ; PubMed Central PMCID: PMCPMC4463005. 25778753 10.1093/cid/civ195 PMC4463005

[26] Lopez T, Moreno M, Salvador F, Zacarias A, Carvalho R, Tomas E, et al Tuberculosis diagnosed in a rural setting in Angola. Accuracy of follow-up sputum smears to predict outcome. Pathog Glob Health. 2013;107(1):5–10. Epub 2013/02/26. 10.1179/2047773212Y.0000000066 ; PubMed Central PMCID: PMCPMC4001596. 23432857 10.1179/2047773212Y.0000000066 PMC4001596

[27] Thompson EG, Du Y, Malherbe ST, Shankar S, Braun J, Valvo J, et al Host blood RNA signatures predict the outcome of tuberculosis treatment. Tuberculosis (Edinb). 2017;107:48–58. Epub 2017/10/21. 10.1016/j.tube.2017.08.004 ; PubMed Central PMCID: PMCPMC5658513. 29050771 10.1016/j.tube.2017.08.004 PMC5658513

