# Supplementary material for: Feature selection and prediction of treatment failure in tuberculosis
Source: PLoS One. 2018 Nov 20;13(11):e0207491. doi: 10.1371/journal.pone.0207491 (PMC6245785; doi:10.1371/journal.pone.0207491)
Supplement: S1 Table — IQR: interquartile range. (DOCX) [file pone.0207491.s001.docx]

**APPENDIX: Supplementary Tables and Figures**

**Table S1.**

| **Variable** | **Entire cohort, N= 587** | **Training, N= 411** | **Testing, N= 176** |
| --- | --- | --- | --- |
| **Median age in years**  **(IQR)** | 40 (30-51) | 40 (29-50) | 42 (31-53) |
| **Sex (n (%))** | Male: 399 (68%)  Female: 188 (32%) | Male: 277 (67.4%)  Female: 134 (32.6%) | Male: 122 (69.3%)  Female: 54 (30.7%) |
| **Treatment failure (n (%))** | 147 (25%) | 103 (25.1%) | 44 (25%) |
| **Patients’ country of origin (n (%))** | Azerbaijan: 51 (8.7%)  Belarus: 412 (70.2%)  Georgia: 54 (9.2%)  Moldova: 17 (2.9%)  Romania: 53 (9%) | Azerbaijan: 34 (8.3%)  Belarus: 289 (70.3%)  Georgia: 39 (9.5%)  Moldova: 15 (3.6%)  Romania: 34 (8.3%) | Azerbaijan: 17 (9.7%)  Belarus: 123 (69.9%)  Georgia: 15 (8.5%)  Moldova: 2 (1.1%)  Romania: 19 (10.8%) |
