# Supplementary material for: Feature selection and prediction of treatment failure in tuberculosis
Source: PLoS One. 2018 Nov 20;13(11):e0207491. doi: 10.1371/journal.pone.0207491 (PMC6245785; doi:10.1371/journal.pone.0207491)
Supplement: S2 Table — Predictive performance is higher than in the complete cases analysis (Table 3). AUC: Area under the receiver-operator curve, PPV: Positive predictive value, NPV: Negative predictive value, LASSO: Least absolute shrinkage and selection operator, SVM: Support vector machine. (DOCX) [file pone.0207491.s002.docx]

**Table S2**

| **Method** | **AUC (95% CI)** | **Misclassi-fication** | **Sensitivity** | **Specificity** | **PPV** | **NPV** |
| --- | --- | --- | --- | --- | --- | --- |
| **Forward stepwise selection** | 0.77 (0.69-0.85) | 0.23 | 0.28 | 0.93 | 0.54 | 0.81 |
| **Backward stepwise elimination** | 0.75 (0.67-0.83) | 0.23 | 0.30 | 0.91 | 0.52 | 0.81 |
| **Backward stepwise elimination & forward stepwise selection** | 0.75 (0.67-0.83) | 0.23 | 0.30 | 0.91 | 0.52 | 0.81 |
| **LASSO** | 0.73 (0.65-0.82) | 0.24 | 0.11 | 0.96 | 0.46 | 0.77 |
| **Random forest** | 0.73 (0.65-0.81) | 0.23 | 0.22 | 0.94 | 0.53 | 0.79 |
| **SVM linear kernel** | 0.71 (0.62-0.79) | 0.25 | 0.11 | 0.95 | 0.42 | 0.77 |
| **SVM polynomial kernel** | 0.70 (0.61-0.78) | 0.24 | 0 | 1 | NA | 0.76 |
