# Supplementary figures and images for: Feature selection and prediction of treatment failure in tuberculosis
Source: PLoS One. 2018 Nov 20;13(11):e0207491. doi: 10.1371/journal.pone.0207491 (PMC6245785; doi:10.1371/journal.pone.0207491)

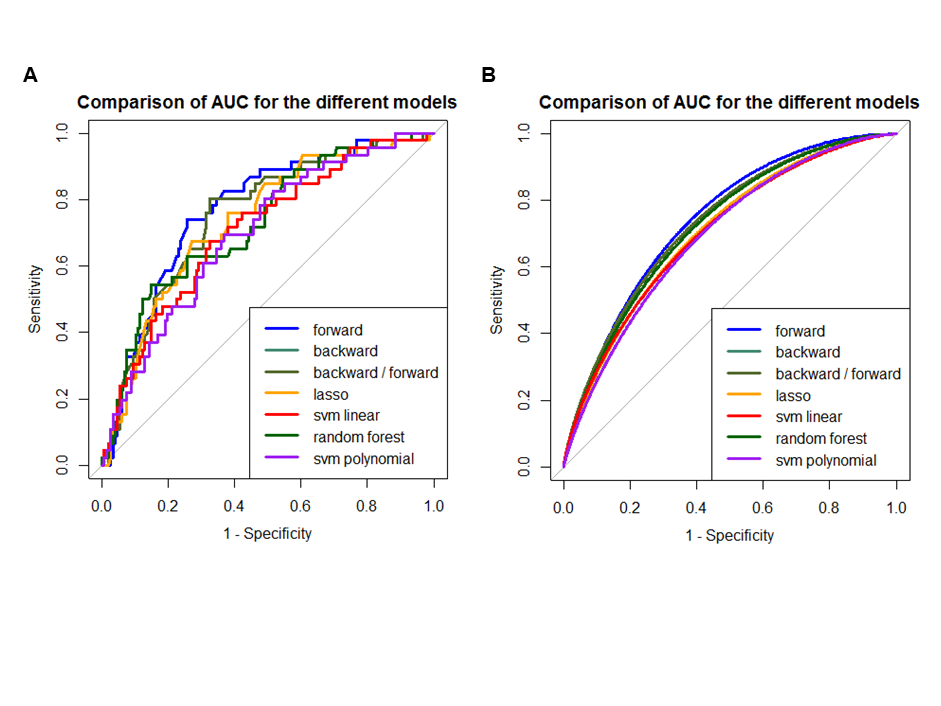

Supplement: S1 Fig — Predicted (A) and smoothed (B) AUC for the different models in the imputed dataset. (TIF) [file pone.0207491.s003.tif]
